# Interfacing With Alpha Motor Neurons in Spinal Cord Injury Patients Receiving Trans-spinal Electrical Stimulation

**DOI:** 10.3389/fneur.2020.00493

**Published:** 2020-06-09

**Authors:** Antonio Gogeascoechea, Alexander Kuck, Edwin van Asseldonk, Francesco Negro, Jan R. Buitenweg, Utku S. Yavuz, Massimo Sartori

**Affiliations:** ^1^Department of Biomechanical Engineering, University of Twente, Enschede, Netherlands; ^2^Department of Clinical and Experimental Sciences, Università degli Studi di Brescia, Brescia, Italy; ^3^Biomedical Signals and Systems Group, University of Twente, Enschede, Netherlands

**Keywords:** alpha motor neuron, coherence, common synaptic input, high-density EMG, spinal cord injury, trans-spinal direct current stimulation, tsDCS

## Abstract

Trans-spinal direct current stimulation (tsDCS) provides a non-invasive, clinically viable approach to potentially restore physiological neuromuscular function after neurological impairment, e.g., spinal cord injury (SCI). Use of tsDCS has been hampered by the inability of delivering stimulation patterns based on the activity of neural targets responsible to motor function, i.e., α-motor neurons (α-MNs). State of the art modeling and experimental techniques do not provide information about how individual α-MNs respond to electrical fields. This is a major element hindering the development of neuro-modulative technologies highly tailored to an individual patient. For the first time, we propose the use of a signal-based approach to infer tsDCS effects on large α-MNs pools in four incomplete SCI individuals. We employ leg muscles spatial sampling and deconvolution of high-density fiber electrical activity to decode accurate α-MNs discharges across multiple lumbosacral segments during isometric plantar flexion sub-maximal contractions. This is done before, immediately after and 30 min after sub-threshold cathodal stimulation. We deliver sham tsDCS as a control measure. First, we propose a new algorithm for removing compromised information from decomposed α-MNs spike trains, thereby enabling robust decomposition and frequency-domain analysis. Second, we propose the analysis of α-MNs spike trains coherence (i.e., frequency-domain) as an indicator of spinal response to tsDCS. Results showed that α-MNs spike trains coherence analysis sensibly varied across stimulation phases. Coherence analyses results suggested that the common synaptic input to α-MNs pools decreased immediately after cathodal tsDCS with a persistent effect after 30 min. Our proposed non-invasive decoding of individual α-MNs behavior may open up new avenues for the design of real-time closed-loop control applications including both transcutaneous and epidural spinal electrical stimulation where stimulation parameters are adjusted on-the-fly.

## 1. Introduction

Spinal cord injury (SCI) disrupts synaptic inputs to below-injury motor, sensory and inter-neurons, thereby impairing physiological sensory-motor function (1). SCI is largely caused by physical trauma to spinal vertebrae, column disks or ligaments following accidents, falls, or sports injuries. Incomplete SCI is the most prevalent type of SCI (2), which causes limb paresis, muscle spasticity, or chronic pain, among other symptoms.

State of the art treatments aim at improving remaining neuromuscular function post-injury via pharmacological therapy (3), stem cell therapy (4), surgical intervention (5), or electrical stimulation. Over the past decade, growing interest has been directed to electrical spinal cord stimulation techniques. Supra-threshold electrical stimulation is used to establish functional neuroprostheses for restoring motor function, with epidural stimulation recently enabling activation of lumbar spinal circuits in rats (6), non-human primates (7) and paraplegic individuals (8). On the other hand, sub-threshold stimulation is used to modulate spinal excitability and induce spinal plastic changes (9–11), rather than establishing functional neuroprostheses. In SCI (12, 13) and stroke (14) patients, sub-threshold stimulation suppressed severe lower limb spasticity and enabled motor control. In this context, neuromodulation of the sub-threshold properties of the spinal neurons (resting potential, excitability) was key to recovery (12). However, while spinal cord stimulation became a standard for treating chronic pain, its use for motor dysfunctions, such as spasticity is still limited and often remains non-clinically accepted (15). With few exceptions (16, 17), spinal cord stimulation techniques operate in “open-loop,” with parameters empirically hand-tuned and with no real-time corrective feedback at the level of α-motor neuron (α-MNs) cellular activity. This is a major element hampering applicability to clinical settings.

Sub-threshold transcutaneous spinal direct current stimulation (tsDCS) in particular, would have large potentials for clinical translation due to its non-invasive and unobstructive nature (18). However, due to the technique inherent non-selectivity as well as spinal cord complex bundle-like organization, it remains challenging to estimate how tsDCS alters spinal neuron function as well as resulting motor function. The ability of estimating how spinal neurons would respond to tsDCS in intact patients *in vivo* would enable a new class of closed-loop techniques, where stimulation parameters could be tuned online to optimally modulate the activity of selected neural targets.

Current approaches to estimate tsDCS neuromodulatory effects include perturbation-based experimental methodologies as well as numerical modeling. While modeling approaches are bound to assumptions as well as to parameter identification and validation challenges, current experimental strategies rely on delivering external stimuli to nerves or muscles to probe (indirectly) α-MN pools excitability (via stretch- or H-reflex, F-wave). Brain stimulation is also used to (indirectly) test corticospinal tract excitability, via transcranial magnetic stimulation (TMS)-induced motor evoked potentials (MEPs). Delivered electromechanical stimuli inherently perturb neuromuscular function, thereby altering physiological motor behavior and preventing translation to real-time closed-loop control applications. Additionally, these traditional experimental techniques cannot provide information on the behavior of individual α-MNs, but only on the global behavior of mixed populations. Therefore, a clinically viable, yet higher-resolution analysis of spinal neurophysiological changes after tsDCS is needed.

Here, we introduce an alternative methodology, with respect to current approaches, based on a direct analysis of α-MN behavior in incomplete SCI patients receiving tsDCS. We propose to use leg muscles as a biological interface to α-MNs. This is possible due to the one-to-one relationship between the action potentials produced in α-MNs and the ones generated in muscles. Thus, high-density surface electromyograms (HD-EMG) recorded from muscles contain neural information that can be derived by means of deconvolution-based blind source separation techniques, such as Convolution Kernel Compensation (CKC).

First, we describe an automated algorithm for assessing the quality of HD-EMG-decomposed α-MN spike trains and for selecting only those that contain physiological neural information, thereby addressing limits in current decomposition techniques. This is a central step as features extracted from α-MN spike trains (both in time and frequency domains) are sensitive to wrongly identified spike trains. Second, we employ coherence analysis to examine how the strength of common synaptic input to α-MN pools modulates in response to tsDCS (19–23).

By these means, our methodology provides an alternative approach to understand the effects of tsDCS on lower limb motor impairment after SCI, which may open up new directions for designing closed-loop neuromodulation techniques.

## 2. Methods

### 2.1. Study Protocol

#### 2.1.1. Participants

Four patients (P1–P4) with chronic incomplete SCI were recruited [age 34–70; walking index for SCI > 1; spinal cord independence measure > 30; and American Spinal Injury Association (ASIA) Impairment Scale (AIS) C or D]. Table 1 shows an overview of each patient characteristics. All participants gave their written informed consent prior to the beginning of the study. The procedures and protocol were approved by the local Ethics Committee of Twente (METC Twente, reference number: NL49561.044.14 / P14-22).

**Table 1:** Overview of patients' characteristics.

**Patient** **ID**	**Age**	**Sex**	**Weight (kg)**	**Height (m)**	**Injury level**	**AIS**	**Time since** **SCI (years)**
P1	62	M	81.5	1.79	C4	D	2
P2	67	M	68	1.77	C4	C	3
P3	34	F	87	1.66	C8	C	4
P4	70	F	67	1.63	C7	D	11

*ID, identification code used for each patient; AIS, American Spinal Injury Association (ASIA) Impairment Scale; SCI, spinal cord injury; M, male; F, female*.

#### 2.1.2. Experimental Procedures

The experimental protocol was designed as a double-blinded, sham-controlled crossover study. The setup consisted of a medical chair in which the patients were seated throughout the experiment. Each patient was tightly strapped to the chair with build-in racing belts, limiting any forward movement of the trunk. The upper leg was fixed at a 90° hip angle by tightly attaching it to a solid frame using leg braces including velcro straps. A force platform (Advanced Mechanical Technology, Inc., Watertown, USA) was used to measure isometric ankle joint plantar-flexion force. The platform was placed close to the chair ensuring that the ankle was positioned firmly with an ankle and knee angles of 90°. Maximum voluntary contractions (MVCs) were measured by asking the patients to generate as much plantarflexion force as possible by contracting only gastrocnemius and soleus muscles for at least 5 s. This was repeated three times with a resting period of 1–2 min between trials. The MVC was defined as the highest force within the three trials.

**Figure 1:**
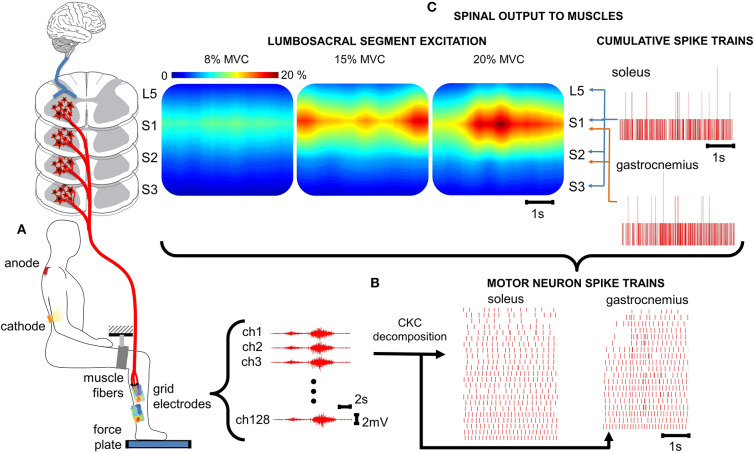
Spatiotemporal spinal maps of ipsilateral α-MNs. **(A)** Experimental set-up for ankle plantar flexion. For the tsDCS, the cathode is placed between the 11th and 12th vertebrae (targeting the lumbosacral spinal segments) and the anode over the right shoulder. HD-EMGs are recorded from the triceps surae and the reference electrode is placed on the malleolus. A brace with velcro closure was attached to immobilize the upper leg and the force plate measures ankle plantar flexion force. **(B)** HD-EMG is decomposed into α-MN spike trains using a convolutive blind-source separation technique (24). **(C)** The spinal output to generate the neural drive to muscles is estimated from the α-MN spike trains. This reveals spatiotemporal information of α-MN activity in the spinal cord across different levels of force (% MVC). % MVC, percentage of maximum voluntary contraction; ch, channel; CKC, convolution kernel compensation.

**Figure 2:**
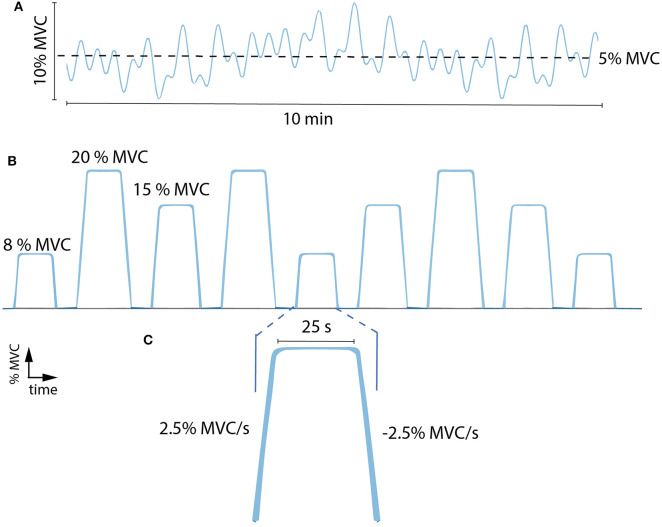
**(A)** Example of a force tracking task during tsDCS. This consisted of following a force trajectory of low-intensity, low-frequency sinusoidal forces with a mean of 5% MVC and a maximum of 10% MVC for a duration of 10 min. **(B)** Example of force tracking tasks during each time condition (pre, t0, t30). These consisted of nine ramp-and-hold sub-maximal plantar flexion contractions at 8, 15, and 20% MVC (i.e., three tasks per level in random order). **(C)** Enlargement of a single force task: ramp up (2.5% MVC/s), hold (25 s) and ramp down (−2.5% MVC/s). % MVC, percentage of maximum voluntary contraction.

Patients underwent two types of stimulation in randomized order: cathodal (2.5 mA) and sham tsDCS. The electrode configuration was the same for both cathodal and sham stimulation: the cathode-electrode was centered between the 11th and the 12th thoracic vertebrae (~L3–L5 segments of the spinal cord, Figures 1A,C) and the anode-electrode was located on the right shoulder (18). During sham, electrical stimulation profiles were ramped up to 2.5 mA and gradually turned off. Stimulation was administered using a custom-build direct-current stimulator (TMS International B.V., Oldenzaal, The Netherlands) while the patients performed the force tracking task for a total period of 15 min including: 8 min of reference force tracking with 3.5 min of rest before and after the tracking exercise. The task consisted of a mixture of sinusoidal waves with a mean of 5% MVC and a maximum of 10% MVC (Figure 2A).

The tsDCS effects were examined at three time conditions including: before (pre), immediately after (t0) and 30 min after (t30) stimulation was delivered. For each condition, patients performed ramp-and-hold tasks that consist of nine sub-maximal plantar flexion contractions at 8, 15, and 20% MVC (i.e., three tasks per level in random order, Figure 2B). Reference and subject-generated force profiles were fed back to the patient via a display. A single task (Figure 2C) consisted of a ramp up (2.5% MVC/s), hold (25 s) and ramp down (2.5% MVC/s).

During each phase, HD-EMGs were recorded using a TMSi Refa multi-channel amplifier (TMS International B.V., Oldenzaal, The Netherlands) with a sampling frequency of 2,048 Hz. A set of two 8x8-electrode grids with 8 and 3 mm inter-electrode distance were placed on the gastrocnemius medialis and soleus muscles, respectively. The grids were applied to the skin using 1-mm thick bi-adhesive foam layer filled with conductive paste to enhance skin-grid contact. The reference electrode is located on the fibula.

### 2.2. Data Analysis

HD-EMG and force data were offline analyzed with Matlab R2019a (The Mathworks Inc., Natick, MA,USA). The HD-EMG recordings were band-pass filtered (50–500 Hz) and decomposed into α-MN spike trains using CKC blind source separation algorithm (25) (Figure 1B). Each α-MN spike train consisted of a vector where the value of 1 indicated the time event at which the respective α-MN fired. The value 0 was used in all time frames where no discharge was detected. Subsequently, cumulative spike trains (CSTs) were defined as the sum of individual α-MN spike trains. CSTs provide the linearity that individual spike trains lack (21) and hence, allow a better estimate of coherence values. Smoothed CSTs were computed using a moving average zero-phase filter with a 400 ms window.

#### 2.2.1. Quality Criteria

This analysis was conducted on soleus and gastrocnemius HD-EMGs recorded from all four patients. As CKC decomposition is a probabilistic iterative procedure to blindly estimate individual spike trains in presence of external noise, errors in the decomposition are inherently expected. Each spike train was inspected for quality control. For this purpose, two quality indices were evaluated in two subsequent steps (see **Algorithm 1**): first, the pulse-to-noise ratio (PNR) and second, the coefficient of variation (CoV) of the inter-spike intervals (ISI).

**Algorithm 1:** Quality control.

**for all** α-MNs in α-MN pool **do**
**STEP 1**: PNR check
**if** PNR < 20 dB **then**
store MN
**else**
remove MN
**end if**
compute *z*_1_
**STEP 2:** CoV_ISI_ {conditional check}
**if** CoV_ISI_ < 0.3 **then**
store MN
**else**
compute *z*_2_ without MN
**if** *z*_2_ > *z*_1_ **then**
remove MN
**else**
store MN
**end if**
**end if**
**end for**

*MN, motor neuron; PNR, pulse-to-noise ratio; *z*_*n*_, z-transformed correlation between force and estimate of neural drive (low-pass filtered cumulative spike train) of the *n*^*th*^ step (*n*=[1,2]); CoV_ISI_, coefficient of variation of ISI (inter-spike intervals)*.

The PNR was defined as the logarithmic ratio (dB) between the means of the innervation pulse train at all time moments in which a α-MN is estimated to have discharged $\left(E({\hat{t}}^{2}(n){|}_{{\hat{t}}^{2}(n)\geq r}\right)$ and not to have discharged $\left(E({\hat{t}}^{2}(n){|}_{{\hat{t}}^{2}(n)<r}\right)$ (24), where $\hat{t}\left(n\right)$ denoted the innervation pulse train as a function of samples *n* and *r* the threshold to detect a pulse (1).

(1) $$\begin{array}{r} PNR(\hat{t}(n))=10\cdot log\left(\frac{E({\hat{t}}^{2}(n){|}_{{\hat{t}}^{2}(n)\geq r}}{E({\hat{t}}^{2}(n){|}_{{\hat{t}}^{2}(n)<r}}\right), \end{array}$$

The coefficient of variation of the ISI was calculated as the ratio between the standard deviation of the ISI and its mean value (20, 25, 26). Only discharges with ISI >33.3 ms (30 Hz) and ISI < 300 ms (3.3 Hz) were included for the CoV_ISI_. Intervals outside this range did not represent physiological values for human leg muscles MNs, thus likely representing decomposition inconsistencies from the CKC algorithm (26–28).

The quality control algorithm computed PNR and CoV sequentially (see **Algorithm 1**). As PNR directly relates to the quality of the decomposition (i.e., ratio between pulse energy and noise level), it was computed as a first filter against low-quality, near-noise spike trains. The algorithm thus rejected all α-MNs with PNRs < 20 dB. The second step verified whether CoV_ISI_ < 0.3 was satisfied (20, 25, 26). Previous motor unit studies showed that motor unit discharge variability increases with SCI (29, 30). Therefore, we computed the Pearson correlation coefficient between reference force profiles and smoothed CSTs (for both soleus and gastrocnemius medialis) before and after the quality criteria were applied and we transformed them into z-scores ($z=\mathrm{arctanh}\sqrt{CPeak}$). The algorithm applied this second condition only if it led to an increase in the correlation between smoothed CST and reference force profile. This was motivated by the fact that, in isometric condition there is direct proportionality between trends in α-MN pool spike trains and resulting muscle force (21).

**Figure 3:**
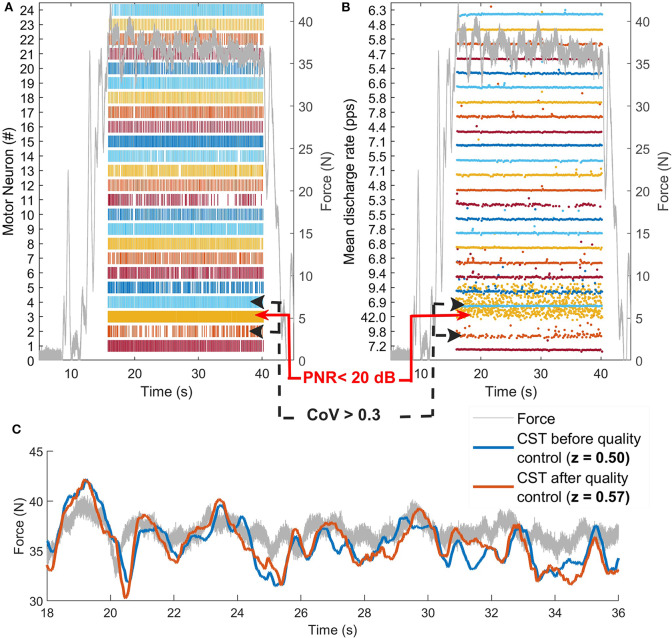
Example of quality control procedure. **(A)** α-MN spike trains and **(B)** discharge rates of a α-MN pool before quality control. The algorithm rejects the α-MN with PNR <20 dB. In this case, as the cross-correlation improved by filtering the α-MNs with a coefficient of variation of the inter-spike intervals >0.3, the algorithm removes them as well. **(C)** Comparison between force (gray line) and the smoothed CST before (blue) and after (red) quality control. For illustrative purposes, the smoothed CST is scaled to the maximum value of the force and shifted to compensate for the neuro-mechanical delay (~0.6 s). Although some spike trains were removed after quality control, the correlation between force and smoothed CST improved. CST, cumulative spike train; pps, pulses per second.

Moreover, because the amount of α-MNs per CST influences the strength of the common synaptic input, α-MN pools with <4 α-MNs were not considered as eligible for quality control and subsequent analyses (section 3). **Algorithm 1** provides the iterative steps computed during the quality selection step. Figure 3 depicts an example of how the quality criteria algorithm works.

#### 2.2.2. Coherence Between CSTs

Because of the presence of pain while undergoing tsDCS, only half of the stimulation intensity (~1.2 mA) was used with P4. For this reason, this analysis was conducted on the data of the soleus muscle only for P1, P2, and P3. For each trial and patient, spike trains decomposed from HD-EMGs were apportioned into three non-intersecting groups and used to create three CSTs. For instance, if the pool contained six α-MN spike trains, three groups with non-intersecting trains were extracted (e.g., α-MN1-α-MN2, α-MN3-α-MN4, and α-MN5-α-MN6).

The magnitude-squared coherence was computed between pairs of detrended CSTs using the Welch's periodogram with Hann windows of 1 s, 50% overlap. Only the steady state interval of both smoothed CSTs was considered. Moreover, the coherence values were transformed into standard Z-scores (31) as follows:

(2) $$\begin{array}{r} COH_{Zscore}=\frac{\mathrm{arctanh}\sqrt{COH}}{\sqrt{1/\left(2N\right)}}, \end{array}$$

where N is the number of segments used to calculate the coherence. The Fisher's z-transform was applied to the coherence values (α = 0.05) in order to compare the normalized coherence values between the conditions (28). Because the low frequencies of common synaptic inputs are associated with force generation (21, 32, 33), significant peaks and areas were extracted in the delta band (<5 Hz). Lastly, we computed the average of the three coherence values between the three groups.

### 2.3. Statistical Analysis

#### 2.3.1. Quality Criteria

**Figure 4:**
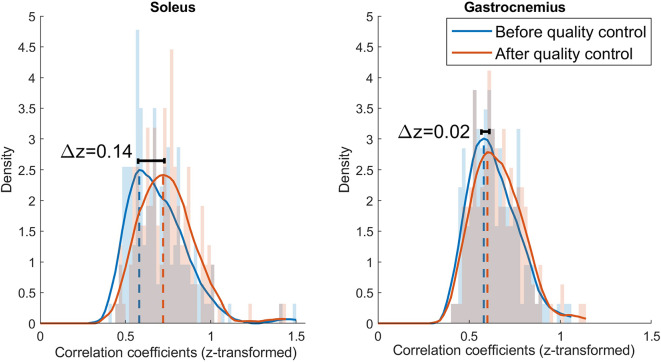
Histograms and kernel density functions for the z-transformed correlation distribution of the soleus and gastrocnemius before and after quality control. The histograms are normalized by the power spectral density and the density function is estimated with a Epanechnikov kernel. The data before quality control is represented in blue and after quality control in orange.

In order to validate the quality control algorithm, the reference force signal and the smoothed CST (neural drive to the soleus and gastrocnemius muscles) were compared for each trial and patient. We assumed that if the correlation between force and CTS signals improved or remained the same after quality control, no relevant neuro-mechanical information was lost. Figure 3C shows how the smoothed CST still reflected the behavior of the reference force profile after some α-MNs with poor quality were removed. In order to compare the z-transformed cross-correlation coefficients at maximum likelihood before-and-after quality control, we computed histograms of the distributions (bin width = 0.02) normalized by the probability density function estimate and fitted into Epanechnikov kernel distributions (Figure 4).

#### 2.3.2. Coherence Analysis

The statistical tests were run using IBM SPSS Statistics v.24 (IBM Corporation, New York, USA). As we recorded nine trials per time condition (three stages: pre, t0, and t30) across sham and cathodal stimulation conditions (i.e., 54 observations per patient), a linear mixed-effects model was performed to include and analyze all the repeated measures for the three patients that entirely followed the protocol.

The time (pre, t0, and t30) and stimulation (cathodal and sham) conditions were defined as fixed effects and the patients were defined as a random effect (i.e., each patient is considered as a group of MN coherence measures). The auto-regressive model AR(1) was specified for the covariance structure of the repeated measures and the models were fitted and compared using the restricted maximum likelihood method. The normality of the residuals was checked with a Shapiro-Wilk test. The significance level was set to 0.05. Linear mixed models do not assume sphericity and are robust against violations of their own assumptions.

## 3. Results

### 3.1. Quality Criteria

We performed a total of 216 HD-EMG recordings per muscle (i.e., soleus and gastrocnemius medialis) across all reference force tracking trials, stimulation conditions and patients. To corroborate the validity of the quality-check algorithm, we only included all cases where α-MN removal was required due to insufficient decomposition quality, i.e., *n* = 157 for the soleus and *n* = 158 for the gastrocnemius medialis, where *n* represents the total number of reference force tracking repetitions.

Figure 3C illustrates a visual example of improvement in correlation before (*z* = 0.50) and after quality control (*z* = 0.57). Table 2 shows the total number of spike trains before and after quality control and the improvement in correlation per subject and muscle. Figure 4 shows the distribution of the z-transformed correlation coefficients (see normalized histograms) as well as the associated kernel probability density functions (maximum likelihood correlation estimator).

**Table 2:** Number of α-MNs before and after quality control and percentage of α-MNs removed for each patient and muscle.

	**Soleus**			**Gastrocnemius**		
	**Number of** **α-MN**			**Number of** **α-MN**		
**Patient** **ID**	**Before QC**	**Affter QC**	**% α-MNs removed**	**Before QC**	**After QC**	**% α-MNs removed**
P1	820	678	17%	809	705	13%
P2	361	291	19%	631	498	21%
P3	884	687	22%	300	220	27%
P4	1,573	801	49%	1,701	1,140	33%
Total	3,638	2,457	32%	3,441	2,563	26%

*ID, identification code used for each patient; MN, Motor neuron; QC, Quality control*.

Results showed that the z-transformed correlation coefficients were distributed toward higher values after quality control for both muscles, i.e., the maximum likelihood correlation coefficient improved from z = 0.58 to z = 0.72 (Δ z = 0.14) for soleus, and from z = 0.58 to z = 0.60 (Δ z = 0.02) for the gastrocnemius medialis.

### 3.2. Coherence Between CSTs

Modulation of coherence between α-MN CSTs was analyzed in the delta band by extracting variations in coherence peak and area across stimulation type (sham, cathodal) and time-since stimulation (pre, t0, t30).

**Figure 5:**
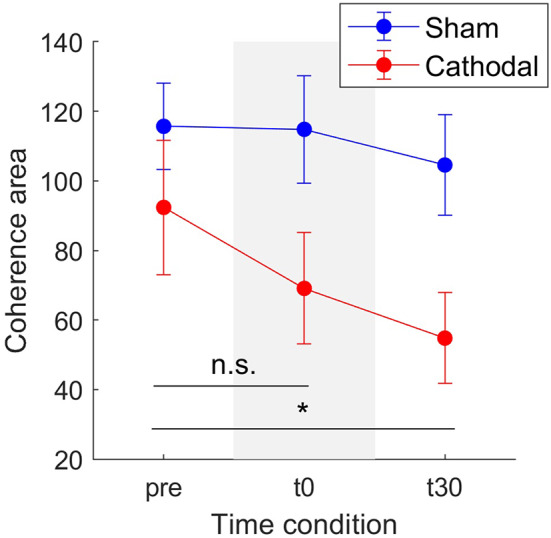
Mean coherence area in the delta band for sham (blue) and cathodal (red) stimulation at pre, just after (t0) and 30 min after (t30) stimulation. (*) indicates significant differences between t30 and pre-stimulation (*p* < 0.05) and n.s. stands for no significance between pre and t0. Error bars represent standard error measure.

Concerning modulation of delta band coherence area, the mixed model analysis showed statistical significant variations for both stimulation type [*F*_(1, 34.58)_ = 23.77, *p* < 0.001] and time-since-stimulation [*F*_(2, 48.04)_ = 4.66, *p* = 0.014] conditions. Figure 5 depicts the coherence area mean and standard errors (SE, 95% confidence interval) across pre, t0, and t30 condition in both sham and cathodal stimulation. In this, estimates of the fixed effects showed significant decrease between the pre- and t30 trials [*t*_(44.92)_ = −3.053, *p* = 0.004]. Mean coherence area decreased from *z*_*pre*_ = 92.36 (SE = 19.35) to *z*_*t*30_ = 69.09 (SE = 16.01) and then to *z*_*t*30_ = 54.8 (SE = 13.05) across time conditions with cathodal stimulation; whereas for sham stimulation, it remained almost constant (*z*_*pre*_ = 115.67, SE = 12.45; *z*_*t*0_ = 114.80, SE = 15.42; *z*_*t*30_ = 104.55, SE = 14.44).

**Figure 6:**
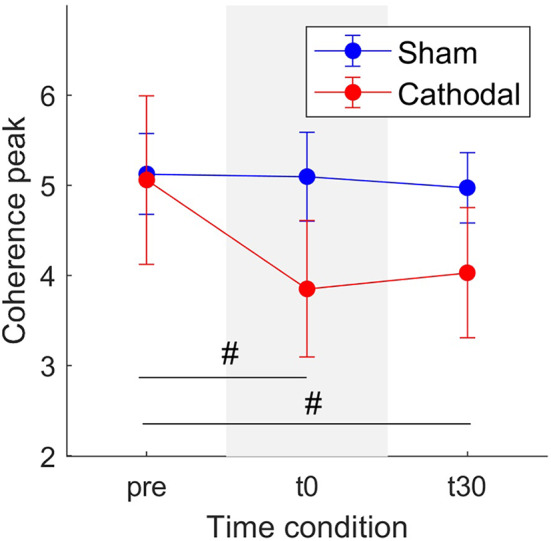
Mean coherence peak in the delta band for sham (blue) and cathodal (red) stimulation at pre, just after (t0) and 30 min after (t30) stimulation. (#) indicates slight evidence of significant differences between pre and t0 and between pre and t30. Error bars represent standard error measure.

Similarly, Figure 6 shows a decrease in coherence peaks from *z*_*pre*_ = 5.06 (SE = 0.93) to *z*_*t*30_ = 3.85 (SE = 0.75) followed by a minimal increase to *z*_*t*30_ = 4.03 (SE =0.72) with cathodal stimulation. For sham stimulation the coherence remained almost constant across all conditions (*z*_*pre*_ = 5.12, SE = 0.45; *z*_*t*0_ = 5.09, SE = 0.49; *z*_*t*30_ = 4.97, SE = 0.39). Nevertheless, in this case, the overall effects were weaker [*F*_(1, 29.92)_ = 3.03, *p* = 0.092] and time [*F*_(2, 45.73)_ = 2.82, *p* = 0.07].

**Figure 7:**
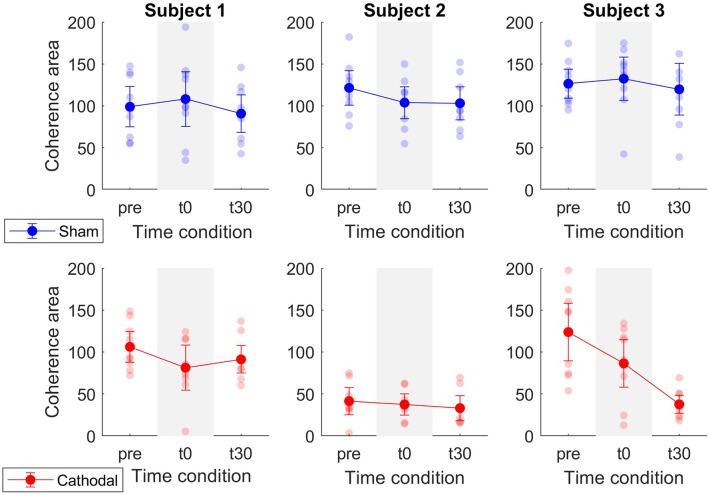
Mean coherence area in the delta band for sham (blue) and cathodal (red) stimulation at pre, just after (t0) and 30 min after (t30) stimulation. Each column depicts the data for each patient.

Patient-specific analyses of both coherence areas and peaks showed pronounced decreasing trends primarily for P1 and P3 (areas: Figure 7 and peaks: Figure 7) for cathodal stimulation. P1 presented a decrease from pre- to t0-stimulation and an slight increase from t0- to t30-stimulation in areas and peaks (areas: *z*_*pre*_ = 106.17, *z*_*t*0_ = 81.29 and *z*_*t*30_ = 91.26; peaks: *z*_*pre*_ = 7.48, *z*_*t*0_ = 5.07 and *z*_*t*30_ = 6.18). Similarly, P3 also presented a decrease in coherence peaks and areas, but with persistent effect across the three time conditions (areas: *z*_*pre*_ = 123.79, *z*_*t*0_ = 86.39 and *z*_*t*30_ = 37.64; peaks: *z*_*pre*_ = 4.68, *z*_*t*0_ = 3.94 and *z*_*t*30_ = 3.05). Contrarily, P2 presented a constant trend during cathodal stimulation (areas: *z*_*pre*_ = 41.48, *z*_*t*0_ = 37.43 and *z*_*t*30_ = 33.08; peaks: *z*_*pre*_ = 2.75, *z*_*t*0_ = 2.53 and *z*_*t*30_ = 2.72). Tables 3, 4 summarize the coherence mean area and peaks across individual patients and conditions.

**Table 3:** Summary of coherence area means and standard errors across patients and experimental conditions.

	**Sham: Mean (SE)**			**Cathodal: Mean (SE)**		
**Patient** **ID**	**pre**	**t0**	**t30**	**pre**	**t0**	**t30**
P1	99.0 (24.1)	108.1 (32.5)	90.8 (22.3)	106.2 (18.3)	81.3 (26.9)	91.3 (16.4)
P2	121.5 (20.6)	103.9 (19.1)	103.1 (19.7)	41.5 (16.1)	37.4 (12.6)	33.1 (14.8)
P3	126.51 (17.34)	132.44 (25.89)	119.80 (30.8)	123.8 (34.3)	86.4 (28.5)	37.6 (10.8)

*ID, identification code used for each patient; SE, Standard error; pre, before stimulation; t0, immediately after stimulation; t30, 30 min after stimulation*.

**Table 4:** Summary of coherence peak means and standard errors across patients and experimental conditions.

	**Sham: Mean (SE)**			**Cathodal: Mean (SE)**		
**Patient** **ID**	**pre**	**t0**	**t30**	**pre**	**t0**	**t30**
P1	4.70 (0.53)	5.66 (1.15)	4.69 (0.69)	7.48 (1.28)	5.07 (1.83)	6.18 (0.89)
P2	5.95 (0.75)	4.89 (0.74)	5.24 (0.64)	2.75 (0.58)	2.53 (0.70)	2.72 (0.69)
P3	4.72 (0.78)	4.74 (0.51)	4.98 (0.72)	4.68 (0.89)	3.94 (0.61)	3.05 (0.34)

*ID, identification code used for each patient; SE, Standard error; pre, before stimulation; t0, immediately after stimulation; t30, 30 min after stimulation*.

## 4. Discussion

This study analyzed for the first time how large populations of α-MNs respond to tsDCS in incomplete SCI individuals performing isometric leg muscle contractions. In this context, we proposed to interface with α-MNs *in vivo*, using decomposition of HD-EMGs and subsequent analysis of α-MN spike trains in the frequency domain (via coherence analysis). Since inter-spike train coherence is sensitive to inaccuracies in the decomposition, we proposed a quality control algorithm for the automatic inspection of individual α-MNs spike trains. With this, we first removed spike trains with a PNR lower than 20 dB. Although a higher threshold for the PNR (30 dB) has been previously used (20, 25), this metric was relative to the quality of the acquired data. We considered that 20 dB (i.e., trains with identified spikes 10 times bigger in average than the baseline) was strict enough as a quality filter avoiding also the loss of relevant information. We then applied a second (conditional) filter using the CoV of the inter-spike intervals. However, as previous studies suggest that α-MN discharge variability increases with SCI (29, 30), this filter was only applied if it improved the correlation between the actual force and the estimate of neural drive (low-pass filtered CST).

We employed coherence analyses to estimate of the strength of the common synaptic input projected onto MS pools as previously proposed (21, 34) and showed that this frequency-dependent feature sensibly responded to stimulation conditions, unlike time-domain features (e.g., discharge rate). With independent synaptic inputs to α-MNs being attenuated by the α-MN pool population sampling, the remaining common synaptic input largely approximates to the neural drive to muscle in the delta band (21) (neural signal for force control). Therefore, coherence and common input analysis may serve as a robust indicator of tsDCS effect on motor function recovery (35). Our results showed consistent decrease in α-MN coherence in delta band immediately after trans-spinal electrical stimulation (i.e., t0-condition) as well as after 30 min from stimulation (i.e., t30-condition) with respect to the non-stimulation condition (i.e., pre-condition, Figures 5, 6). From a neurophysiological point of view, the observed coherence decrease may underlie a decrease in the strength of common synaptic input to α-MN pools. Previous research (34) indicated that de-correlation between α-MN spike trains may be due to additional components of the common synaptic input to all α-MNs, but independent to the cortical drive. Thus, the reduction in delta band coherence may indicate demodulation (increased variability) in effective neural drive to the muscle, possibly due to higher frequency inputs.

**Figure 8:**
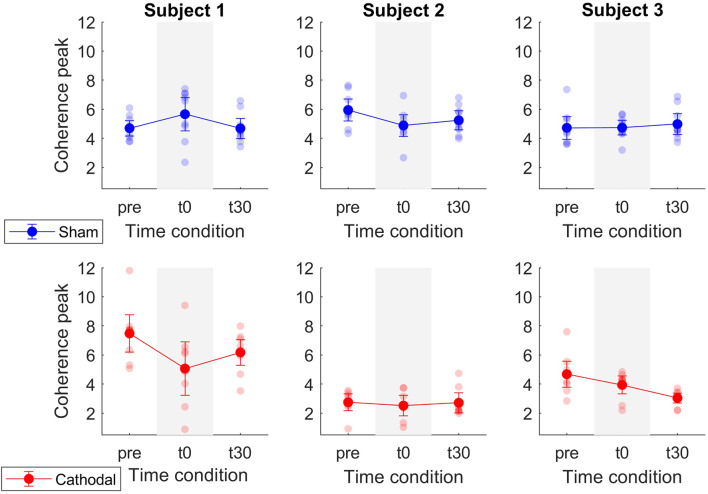
Mean coherence peak in the delta band for sham (blue) and cathodal (red) stimulation at pre, just after (t0) and 30 min after (t30) stimulation. Each column depicts the data for each patient.

Coherence peak and area modulations showed less pronounced trends in P2 with respect to P1 and P3 (Figures 7, 8). That is, average coherence peaks and areas for P2 did not vary substantially across pre-, t0-, and t30-conditions. This may be due to the inability of personalizing the stimulation parameter for the individual patient (i.e., stimulation intensity) as well as the inability of accurately targeting the desired spinal segments (~L5-S2). Moreover, it is worth stressing that tsDCS was prescribed blindly with no feedback on α-MN behavior. In this context, lesion and spinal cord anatomy differences across patients could explain the variability in the stimulation-effect (cathodal) results. Although, in this study, the lesion differences are rather small [all patients have injuries above C8 and their motor function is preserved below the injury (AIS-C and D)], larger differences can be found in the patients' physiological and anatomical factors including age, sex, height, and weight (Table 1). Future work will develop on-line HD-EMG recording and decomposition techniques to be used to adjust on the fly the location and stimulation parameters of multi-channel electrical stimulation.

We analyzed changes in α-MN coherence features with no direct observation of stimulation-effects on the neuromuscular system. Future work will employ electrically induced H-reflex to verify whether or not the administered stimulation induced facilitation of corticospinal output to muscles, thus providing further possibilities for interpreting coherence results at the α-MN level.

Moreover, the present study aimed at creating a methodology to analyze for the first time the response of multiple MNs to tsDCS in incomplete SCI individuals. However, further clinical validation is needed to test whether our methodology can be generalized to a larger population. Future work will extend this study to also include healthy individuals, multiple muscles and data modeling approaches.

The quality-check algorithm we proposed (**Algorithm 1**, Figure 3) may also be further improved in the future to enable detailed editing of α-MN spike trains. Our proposed methodology only enabled removing entire spike trains after inconsistencies being detected. This may result in discarding potentially neurophysiologically-consistent information. The ability of identifying individual mismatched spikes (36) would avoid the need for eliminating entire spike trains in the quality control stage. As a result, less neural information would be compromised. However, this would make the experimental set-up more complicated as it requires not only surface EMG but also intramuscular EMG recordings.

This study demonstrated the ability of interfacing with α-MNs *in vivo* in SCI individuals receiving transcutaneous spinal cord electrical stimulation. First, we proposed a methodology that enabled removing compromised information from α-MN spike trains, central for analysing frequency domain features from bio-electrical data. Second, we reported how α-MN discharge coherence modulated in response to spinal cord electrical stimulation. Our study suggested that the common synaptic input to α-MN pools may decrease immediately after cathodal tsDCS, which was reflected in the decrease in coherence within the delta band.

The ability to non-invasively estimate how α-MNs respond to electrical stimuli is central to devise personalized rehabilitation therapies. To this aim, future work will first establish causal relations between common synaptic input to α-MNs and spinal cord excitability. In a rehabilitation scenario, knowing these relationships will enable building direct associations between electrical stimulation patterns and the resulting modulation in an individual patient's spinal cord excitability. This will permit using non-invasive electrical stimulation to restore physiological spinal excitability for treating a variety of related motor disorders associated with hyperexcitability of spinal neuronal structures, such as spasticity (37). Moreover, the ability to modulate spinal cord excitability may help understand how to best induce activity-dependent neuro-plasticity, thereby increasing the efficacy of rehabilitation programs (38).

In the context of neurorehabilitation technologies, our proposed methodology may open up new avenues for the design of real-time model-based closed-loop applications (39–41) including both transcutaneous and epidural spinal cord electrical stimulation methodologies. To this end, non-invasive estimates of individual spinal α-MN behavior may help optimize stimulation parameters on-the-fly (e.g., pulse width and amplitude of a multi-channel array of electrodes), thereby modulating spinal excitability in a closed-loop fashion.

## Data Availability Statement

The datasets generated for this study are available on request to the corresponding author.

## Ethics Statement

The studies involving human participants were reviewed and approved by Ethics Committee of Twente (Enschede, The Netherlands). The patients/participants provided their written informed consent to participate in this study.

## Author Contributions

AG, AK, EA, FN, JB, UY, and MS contributed to the revision of the manuscript, approved it for submission, and agreed to be accountable for all aspects of the work in ensuring that questions related to the accuracy or integrity of any part of the work are appropriately investigated and resolved. AK and EA contributed to the data acquisition. AG, FN, JB, UY, and MS contributed to the data analysis and interpretation. AG, UY, and MS contributed to the conception of the work and writing of the manuscript.

## Conflict of Interest

The authors declare that the research was conducted in the absence of any commercial or financial relationships that could be construed as a potential conflict of interest.
